# Transcriptomic analysis of expression of genes regulating cell cycle progression in porcine ovarian granulosa cells during short-term in vitro primary culture

**DOI:** 10.1007/s00418-020-01860-2

**Published:** 2020-03-10

**Authors:** Magdalena Kulus, Wiesława Kranc, Patrycja Sujka-Kordowska, Piotr Celichowski, Aneta Konwerska, Maurycy Jankowski, Michal Jeseta, Mariusz T. Skowroński, Hanna Piotrowska-Kempisty, Dorota Bukowska, Maciej Zabel, Małgorzata Bruska, Paul Mozdziak, Bartosz Kempisty, Paweł Antosik

**Affiliations:** 1 Department of Veterinary Surgery, Institute of Veterinary Medicine, Nicolaus Copernicus University in Torun, Toruń, Poland; 2 Department of Anatomy, Poznan University of Medical Sciences, 6 Święcickiego St., 60-781 Poznan, Poland; 3 Department of Histology and Embryology, Poznan University of Medical Sciences, 6 Święcickiego St., 60-781 Poznan, Poland; 4 Department of Obstetrics and Gynecology, University Hospital and Masaryk University, Brno, Czech Republic; 5 Department of Basic and Preclinical Sciences, Institute of Veterinary Medicine, Nicolaus Copernicus University in Torun, Toruń, Poland; 6 Department of Toxicology, Poznan University of Medical Sciences, Poznan, Poland; 7 Department of Diagnostics and Clinical Sciences, Institute of Veterinary Medicine, Nicolaus Copernicus University in Torun, Toruń, Poland; 8 Department of Histology and Embryology, Wroclaw Medical University, Wrocław, Poland; 9 Division of Anatomy and Histology, University of Zielona Gora, Zielona Gora, Poland; 10 Physiology Graduate Program, North Carolina State University, Raleigh, NC USA

**Keywords:** Pig, Ovarian follicle, Granulosa cells, Primary culture, Microarray

## Abstract

The primary function of ovarian granulosa cells (GCs) is the support of oocytes during maturation and development. Molecular analyses of granulosa cell-associated processes, leading to improvement of understanding of the cell cycle events during the formation of ovarian follicles (folliculogenesis), may be key to improve the in vitro fertilization procedures. Primary in vitro culture of porcine GCs was employed to examine the changes in the transcriptomic profile of genes belonging to “cell cycle”, “cell division”, “cell cycle process”, “cell cycle phase transition”, “cell cycle G1/S phase transition”, “cell cycle G2/M phase transition” and “cell cycle checkpoint” ontology groups. During the analysis, microarrays were employed to study the transcriptome of GCs, analyzing the total RNA of cells from specific periods of in vitro cultures. This research was based on material obtained from 40 landrace gilts of similar weight, age and the same living conditions. RNA was isolated at specific timeframes: before the culture was established (0 h) and after 48 h, 96 h and 144 h in vitro. Out of 133 differentially expressed genes, we chose the 10 most up-regulated (*SFRP2*, *PDPN*, *PDE3A*, *FGFR2*, *PLK2*, *THBS1*, *ETS1*, *LIF*, *ANXA1*, *TGFB1*) and the 10 most downregulated (*IGF1*, *NCAPD2*, *CABLES1*, *H1FOO*, *NEK2*, *PPAT*, *TXNIP*, *NUP210*, *RGS2* and *CCNE2*). Some of these genes known to play key roles in the regulation of correct cell cycle passage (up-regulated SFRP2, PDE3A, PLK2, LIF and down-regulated CCNE2, TXNIP, NEK2). The data obtained provide a potential reference for studies on the process of mammalian folliculogenesis, as well as suggests possible new genetic markers for cell cycle progress in in vitro cultured porcine granulosa cells.

## Introduction

During the life cycle, cells go through distinct successive stages, which have been divided into phases. The order in which they occur, as well as their correctness are determined primarily by expression of a particular set of genes. Abnormalities in the passage of individual phases lead to the formation of abnormal cells, including those that give rise to a variety of cancers (Bertoli et al. 2013). Specific “checkpoints”, verify progression at each stage of the cell cycle, controlling the DNA integrity. These mechanisms are able to detect damaged DNA and/or replication errors, excluding these fragments from further activity or arresting the cycle, which prevents the formation of abnormal cells (Bertoli et al. 2013). The importance of these checkpoints is evidenced by significant percentage of mutations within their regulatory proteins leading to the onset of oncogenesis. Cells in the G1 phase of the cell cycle need process a number of information governing the transition to the next phase-S-replication. Errors in the processes of growth, proliferation and stress management in the G1 phase were found to lead to the formation of cancers (Massagué 2004). Through an in-depth analysis of molecular processes of these critical cell cycle stages, it may be possible to identify particular causes of oncogenesis and provide a basic reference for the potential development of targeted therapies.

In a mature ovarian follicle, two walls of ovarian granulosa cells surround the fluid filled cavity and the developing oocyte (Budna et al. 2017; Rybska et al. 2018a). The female gamete is surrounded directly by a layer of cumulus cells (CCs), with the oocyte adjacent portion referred to as corona radiata. The second layer, lining the inside of the follicle, are the mural granulosa cells (GCs), surrounded from the outside by a basement membrane. Finally, the outermost cells building the ovarian follicles are theca cells (TCs). All types of ovarian granulosa play a very important physiological role in the follicular function. TCs produce androgenic substrate, necessary for the production of estrogen (Gilchrist et al. 2004; Magoffin 2005). CCs belonging to corona radiata adhere to the zona pellucida, a glycoprotein capsule penetrated by microvilli from both the oocyte and the cumulus cells. This process facilitates communication between these cells, conducted via ions and molecules exchanged using gap-junction nexus connections (Kempisty et al. 2014). Finally, GCs play a role in the formation of the corpus luteum after ovulation, consequently participating in progesterone production (Chermuła et al. 2019).

Furthermore, mammalian ovaries are a rich source of cells that can be used for molecular research. The granulosa cells (GCs) of the ovarian follicle are an interesting example, being able to proliferate in primary in vitro cultures despite the lack of their usual physiological environment, with recent findings indicating many possibilities for their application in molecular studies (Kranc et al. 2015, 2016). Molecular analysis of the basic granulosa cell-associated processes, as well as understanding of their cell cycle events during the formation of ovarian follicles (folliculogenesis) may be a key factor to improve the in vitro fertilization procedures, providing us with new molecular markers indicating the correctness of the folliculogenesis process, as well as oocyte development and maturation (Kranc et al. 2018; Rybska et al. 2018b).

Additionally, ovarian granulosa cells show significant stem-like potential. Literature data indicates that female GCs expressed molecular markers that are characteristic for mesenchymal stem cells (e.g. CD29, CD44, CD105, CD90) or pluripotent stem cells (Oct4, Nanog, Sox2, Tert) (Kossowska-Tomaszczuk et al. 2009, 2010). Additionally, other studies have shown that GCs may differentiate into osteoblasts (Mattioli et al. 2012) and chondrocytes (Varras et al. 2012). Thanks to the characteristics shown by these cells and relatively simple methods of obtaining them, they may become a model for developing stem cell therapies, with potential application in fields such as regenerative medicine. However, it is first necessary to fully describe the molecular basis of their functioning in in vitro culture, including the genes responsible for the cell cycle, which will allow for their further in vivo study and potential applications in clinical situations.

The objective of the current study was to study transcriptomic profiling of in vitro cultured porcine GCs. The focus was placed on transcriptomic profile alterations of genes belonging to “cell cycle”, “cell division”, “cell cycle process”, “cell cycle phase transition”, “cell cycle G1/S phase transition”, “cell cycle G2/M phase transition” and “cell cycle checkpoint” ontology group. The knowledge gained should help to improve the understanding of GC functioning in in vitro conditions, as well as provide a basic molecular reference for further in vivo studies that could possibly lead to application of ovarian granulosa in clinical situations, as well as improve and optimized the currently used IVF techniques.

## Materials and methods

### Animals

Samples were obtained from 40 crossbred landrace gilts, all kept in the same conditions (feed, breeding, housing). These pigs had a mean weight of 98 kg and age of 170 days. The experiment was approved by the Poznan University of Medical Sciences Bioethical Committee (Resolution 32/2012, approved 1st of June 2012).

### Collection of porcine ovaries and in vitro culture of granulosa cells

The reproductive organs were transported to the laboratory under appropriate temperature conditions (38 °C) in 0.9% NaCl within 30 min of slaughter. Then, the ovaries (80 in total) of the individual animals were isolated and placed in PBS supplemented with fetal bovine serum (FBS; Sigma-Aldrich Co., St. Louis, MO, USA). Large pre-ovulatory follicles with diameters above 5 mm (*n* = 300) were selected. Individual follicles were punctured with a sterile 20-G needle and aspirated with a 5 ml syringe. The procedure was performed in a Petri dish, recovering cumulus-oocyte complexes (COCs) and follicular fluid (FF). Subsequently, GCs were obtained while COCs were discarded.

The obtained cells were suspended in the culture medium and counted using ADAM Cell Counter and Viability Analyzer (Bulldog Bio, Portsmouth, NH, USA) and seeded on specific culture vessels. In this study, a medium of the following composition was used: Dulbecco’s Modified Eagle’s Medium (DMEM, Sigma-Aldrich, USA), 2% fetal calf serum (FCS) (PAA, Linz, Austria), 10 mg/ml ascorbic acid (Sigma-Aldrich, USA), 0.05 μM dexamethasone (Sigma-Aldrich, USA), 4 mM l-glutamine (Invitrogen, USA), 10 mg/ml gentamycin (Invitrogen, USA), 10,000 units/ml penicillin and 10,000 μg/ml streptomycin (Invitrogen, USA).

Stable conditions of 38.5 °C and 5% CO_2_ were maintained during the culture of the obtained cells. At the indicated time intervals of the culture: 0 h, 24 h, 48 h and 96 h, GCs separated from the bottom of the culture dish using 0.05% trypsin–EDTA (Invitrogen, USA) for 3 min. To count the cells in the samples, an ADAM Cell Counter and Viability Analyzer (Bulldog Bio, Portsmouth, NH, USA) were used.

### Microarray expression analysis and statistics

The Affymetrix procedure was previously described by Trejter et al. (2015). cDNA was reverse transcribed from the Total RNA of each sample (100 ng) (Ambion^®^ WT Expression Kit). Obtained cDNA was biotin labeled and fragmentated using Affymetrix GeneChip^®^ WT Terminal Labeling and Hybridization. Biotin-labeled fragments of cDNA (5.5 μg) were hybridized to the Affymetrix^®^ Porcine Gene 1.1 ST Array Strip (48 °C/20 h). Then, the microarrays were washed and stained according to the technical protocol of the Affymetrix GeneAtlas Fluidics Station. Subsequently, the array strips were scanned by the Imaging Station of the GeneAtlas System. The preliminary analysis of the scanned chips was performed using the Affymetrix GeneAtlas™ Operating Software. The quality of gene expression data was checked according to quality control criteria provided by the software. Obtained CEL files were imported into the downstream data analysis software. All of the presented analyses and graphs were compiled using Bioconductor and R programming language. Each CEL file was merged with a description file. To correct background, normalize and summarize results, a Robust Multiarray Averaging (RMA) algorithm was employed.

Statistical significance of the analyzed genes was conducted using moderated *t*-statistics from the empirical Bayes method. Obtained *p* value was corrected for multiple comparisons using the Benjamini and Hochberg’s false discovery rate. The selection of significantly changed gene expression was based on a *p* value beneath 0.05 and expression fold higher than 2. Differentially expressed genes were subjected to the selection of genes associated with cell cycle progression. Differentially expressed gene lists (separate for up and down regulated groups) were uploaded to the DAVID software (Database for Annotation, Visualization and Integrated Discovery), with enriched Gene Ontology terms extracted. Among the Enriched Gene Ontology terms, we have chosen those containing at least 5 genes and exhibiting a Benjamini method calculated *p* value lower than 0.05. Among the enriched Gene Ontology terms, we have chosen “cell cycle checkpoint”, “cell cycle G1/S phase transition”, “cell cycle G2/M phase transition”, “cell cycle phase transition”, “cell cycle process”, “cell cycle” and “cell division” Gene Ontology Biological Process (GO BP) terms. Expression data of genes within the selected GO BP terms were subjected to hierarchical clusterization procedure and presented as heatmaps.

To further analyze the chosen gene sets, we investigated their mutual relations using the GOplot package (Walter et al. 2015). Moreover, the GOplot package was used to calculate the *z*-score (the number of up-regulated genes minus the number of down-regulated genes divided by the square root of the count). *z*-Score analysis allowed us to compare the enrichment of the selected GO BP terms.

Moreover, the interactions between proteins coded by selected genes and the genes itself were investigated using the STRING10 software (Search Tool for the Retrieval of Interacting Genes). STRING database contains information on protein/gene interactions, including experimental data, computational prediction methods and public text collections. STRING database engine provided us with a molecular interaction network formed between the genes of interest. The search criteria are based on co-occurrences of genes/proteins in scientific texts (textmining), co-expression and experimentally observed interactions.

Finally, the functional interactions between the genes belonging to the chosen GO BP terms were investigated using the REACTOME FIViz application to the Cytoscape 3.6.0 software. The ReactomeFIViz app is designed to find pathways and network patterns related to cancer and other types of diseases. This app accesses the pathways stored in the Reactome database, allowing to perform pathway enrichment analysis for a set of genes, visualize hit pathways using manually laid-out pathway diagrams directly in Cytoscape, and investigate functional relationships among genes in hit pathways. It can also access the Reactome Functional Interaction (FI) network, a highly reliable, manually curated pathway-based protein functional interaction network covering over 60% of human proteins.

### RT-qPCR validation

Total RNA was isolated from GCs after 0 h, 24 h, 48 h and 96 h of in vitro culture using the Chomczyński and Sacchi method (Chomczynski and Sacchi 1987; Borys-Wójcik et al. 2018; Chamier-Gliszczyńska et al. 2018). The RNA samples were resuspended in 20 µl of RNase-free water and stored in liquid nitrogen. The samples were treated with DNase I and reverse-transcribed (RT) into cDNA. RT-qPCR was conducted in a LightCycler real-time PCR detection system (Roche Diagnostics GmbH, Mannheim, Germany) using SYBR^®^ Green I as a detection dye. Target cDNA was quantified using the relative quantification method. The relative abundance of the analyzed transcripts in each sample was standardized to the internal standards. For amplification, 1 µl of cDNA solution was added to 9 µl of QuantiTect^®^ SYBR^®^ Green PCR (Master Mix Qiagen GmbH, Hilden, Germany) and primers (Table 1). Each experiment was performed in 3 biological and three technical replicates.

**Table 1:** Oligonucleotide sequences of primers used to conduct the RT-qPCR reactions

Gene	Gene ID	Primer sequence (5′–3′)
*CCNE2*	9134	**F**: GATGGTGCTTGCAGTGAAGA
		**R**: CGATGGCTAGAATGCACAGA
*RGS2*	5997	**F**: CTAAGGCGGTCCAATCACAT
		**R**: GCCCTCAAAAGACAGCAGAC
*NUP210*	23225	**F**: GCAACTGAAGCACCTGAACA
		**R**: ATGGCACCAAAGACCTTGAC
*TXNIP*	10628	**F**: CAAGCCAGCCAACTCAAGAG
		**R**: TTCGAGCAGAGACAGACACC
*PPAT*	5471	**F**: ACCGTGAAGTCTTACCTGGA
		**R**: TCGAAGATACAGAAAGCCATTGG
*NEK2*	4751	**F**: TGGGAAGATCAGAGAAGGCA
		**R**: TGGAGTCCTGCAGCTTTTCT
*H1FOO*	132243	**F**: CAGTCACCTCCCAGAACCAC
		**R**: TGTTCCCCATCTTCGTTTTGG
*CABLES1*	91768	**F**: CGTCGTCTCATCTCCCAGAG
		**R**: CATTCCTGGTGTCGTGCTG
*NCAPD2*	9918	**F**: CATTTCAGGCTGCCTTTCGA
		**R**: CTGGGAGTGGCGGGATAC
*IGF1*	3479	**F**: TTCTACTTGGCCCTGTGCTT
		**R**: CTCCAGCCTCCTCAGATCAC
*TGFB1*	7040	**F**: AAGCGGCAACCAAATCTATG
		**R**: CACGTGCTGCTCCACTTTTA
*ANXA1*	301	**F**: GGCCTTGGAACTGATGAAGA
		**R**: CCTCAGATCGGTCACCCTTA
*LIF*	3976	**F**: GAGGGAACCCAGAGTCTTCC
		**R**: TAGCACTGCTGGATGTCAGG
*ETS1*	2113	**F**: CATTGAGCGAGGTGAAGACA
		**R**: TCTGCCTTTGCTTTCCAAGT
*THBS1*	7057	**F**: CAAAGAGTTGGCCAGTGAGC
		**R**: ATGATGGGGCAGGACACTTT
*PLK2*	10769	**F**: CTTCGGGTACCAGCTCTCAG
		**R**: TAGGCAGATCTCCACCATCC
*FGFR2*	2263	**F**: GATGCCATCTCGTCCGGA
		**R**: TGGACAGCGGAACTTGACA
*PDE3A*	5139	**F**: ATGAGGCACCTTCATCCAGT
		**R**: TTCACTAGAGAACCCGGTCG
*PDPN*	10630	**F**: AATGTGGAAGGTGCCAGTTC
		**R**: TTCGTGGGGTCACTGTGTAA
*SFRP2*	6423	**F**: GGAAGAGGGACACTCATGGA
		**R**: TGATTGGAAAGGGAGCATTC

*F* forward primer, *R* reverse primer

One RNA sample of each preparation was processed without the RT-reaction to provide a negative control for subsequent PCR.

To quantify the specific genes expressed in the GCs, the expression levels of specific mRNAs in each sample were calculated relative to PBGD and ACTB. To ensure the integrity of these results, an additional housekeeping gene, 18S rRNA, was used as an internal standard to demonstrate that PBGD and ACTB mRNAs were not differentially regulated in GC groups. 18S rRNA has been identified as an appropriate housekeeping gene for use in quantitative PCR studies. Again, the statistical significance of the analyzed genes was performed using moderated *t*-statistics from the empirical Bayes method. The obtained *p* value was corrected for multiple comparisons using the Benjamini and Hochberg’s false discovery rate.

### Histological examination

Histological examination was performed on ovaries and separated follicles. For this purpose, 3 whole ovaries were collected, with a dozen follicles isolated from 2 ovaries. Immediately after collection, the organs were fixed in Bouin’s solution for 48 h. Subsequently, ovaries and follicles were embedded in paraffin and then cut into 4 μm thick sections with a semi-automatic rotary microtome (Leica RM 2145, Leica Microsystems, Nussloch, Germany). Then, the sections were stained with routine hematoxylin and eosin (H&E) staining method, following the protocol of deparaffinization and rehydration, H&E staining and dehydration. Finally, histological sections were evaluated under light microscope and selected pictures were taken with the use of high-resolution scanning technique and Olympus BX61VS microscope scanner (Olympus, Tokyo, Japan).

## Results

Whole transcriptome profiling with Affymetrix microarrays allowed to analyze the granulosa gene expression changes at 48, 96 and 144 h of in vitro culture, with 0 h sample serving as an entry point reference. With the use of Affymetrix^®^ Porcine Gene 1.1 ST Array Strip, the expression of 27,558 transcripts was examined. Genes with fold change higher than abs (2) and with corrected *p* value lower than 0.05 were considered as differentially expressed. This set of genes consists of 3380 different transcripts, the complete list of which can be found in the GEO database (ID: GSE134361).

**Fig. 1:**
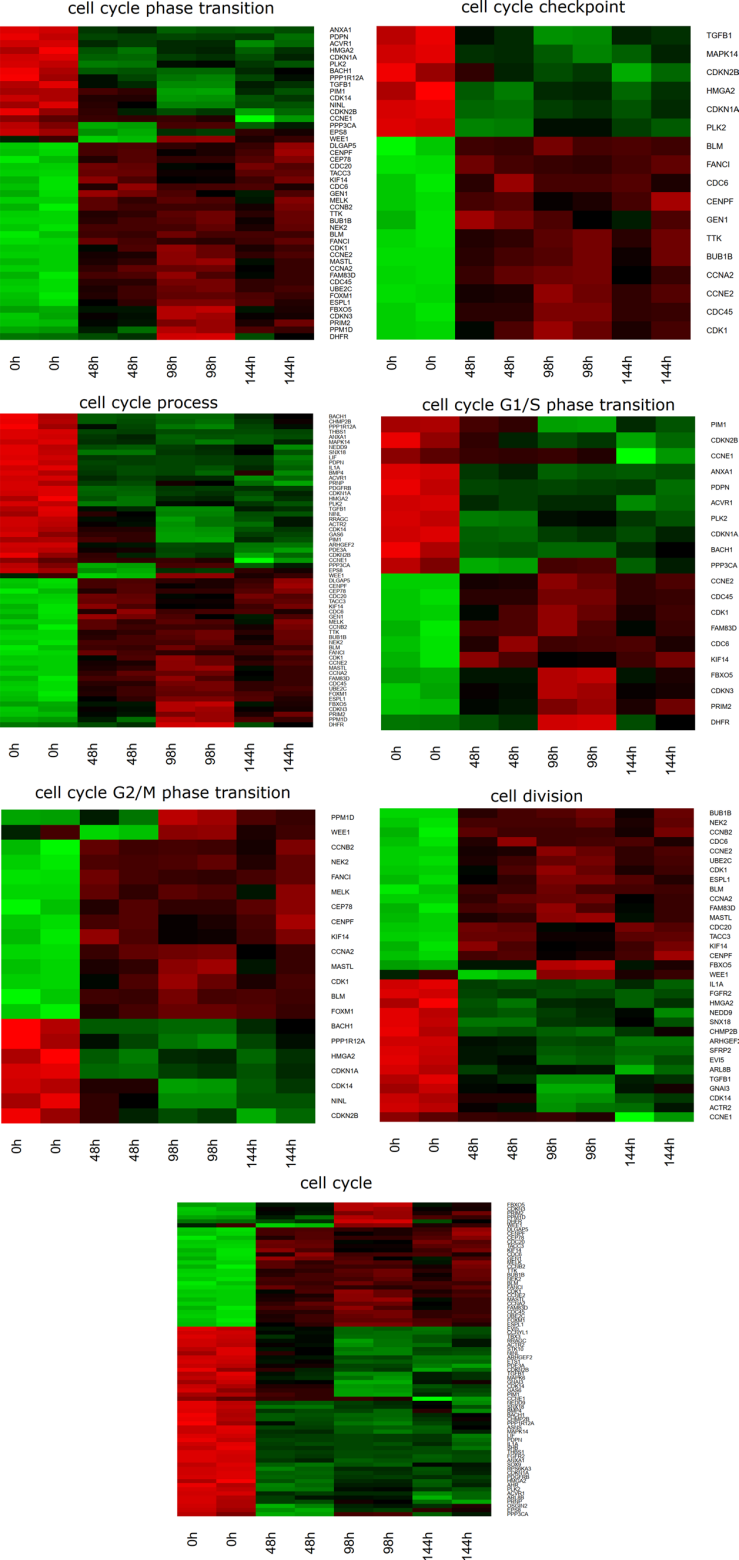
Heat map representation of differentially expressed genes belonging to the “cell cycle checkpoint”, “cell cycle G1/S phase transition”, “cell cycle G2/M phase transition”, “cell cycle phase transition”, “cell cycle process”, “cell cycle” and “cell division” GO BP terms. Arbitrary signal intensity acquired from microarray analysis is represented by colors (green, higher; red, lower expression). log2 signal intensity values for any single gene were resized to Row *z*-score scale (from − 2, the lowest expression to + 2, the highest expression for single gene)

Up and down-regulated gene sets were subjected to the Database for Annotation, Visualization and Integrated Discovery (DAVID) search separately and only ones with an adj. *p* value lower than 0.05 were selected. The DAVID software analysis showed that the differently expressed genes belonged to 344 GO BP Terms. In this paper we focused on “cell cycle checkpoint”, “cell cycle G1/S phase transition”, “cell cycle G2/M phase transition”, “cell cycle phase transition”, “cell cycle process”, “cell cycle” and “cell division” GO BP terms. These sets of genes were subjected to a hierarchical clusterization procedure and presented as heatmaps (Fig. 1). The gene symbols, fold changes in expression, Entrez gene IDs and corrected *p* values of that genes are shown in Table 2.

**Table 2:** Fold rations and *p* values of differentially expressed genes of interest analysed in this study

Gene	Gene ID	Fold ratio 0H/48H	Fold ratio 0H/96H	Fold ratio 0H/144H	*p* value 0H/48H	*p* value 0H/96H	*p* value 0H/144H
*CCNE2*	9134	− 1.192E+01	− 3.423E+01	− 1.777E+01	9.34E−06	9.04E−07	2.79E−06
*RGS2*	5997	− 3.630E+00	− 2.973E+01	− 2.829E+01	1.15E−04	5.23E−07	6.54E−07
*NUP210*	23225	− 6.899E+00	− 2.075E+01	− 1.881E+01	8.07E−04	3.78E−05	6.28E−05
*TXNIP*	10628	− 2.702E+00	− 3.410E+01	− 4.530E+00	1.58E−03	1.12E−06	1.25E−04
*PPAT*	5471	− 4.889E+00	− 1.596E+01	− 9.949E+00	5.19E−05	1.56E−06	5.23E−06
*NEK2*	4751	− 6.253E+00	− 6.790E+00	− 6.013E+00	4.01E−05	1.64E−05	3.12E−05
*H1FOO*	132243	− 5.181E+00	− 5.013E+00	− 6.962E+00	5.30E−06	2.60E−06	1.41E−06
*CABLES1*	91768	− 5.840E+00	− 4.871E+00	− 6.439E+00	1.41E−05	1.21E−05	7.03E−06
*NCAPD2*	9918	− 4.661E+00	− 6.661E+00	− 5.229E+00	1.93E−05	3.24E−06	8.81E−06
*IGF1*	3479	− 8.344E+00	− 5.532E+00	− 2.555E+00	9.96E−05	1.72E−04	6.28E−03
*TGFB1*	7040	4.842E+00	1.036E+01	5.224E+00	3.48E−05	2.35E−06	1.86E−05
*ANXA1*	301	5.681E+00	7.665E+00	7.361E+00	5.56E−06	1.17E−06	1.63E−06
*LIF*	3976	8.345E+00	8.656E+00	1.079E+01	2.91E−05	1.36E−05	1.08E−05
*ETS1*	2113	5.431E+00	1.067E+01	1.223E+01	6.20E−06	6.41E−07	5.91E−07
*THBS1*	7057	9.549E+00	1.038E+01	9.767E+00	9.81E−07	3.32E−07	4.37E−07
*PLK2*	10769	1.894E+01	7.062E+00	1.203E+01	1.08E−06	3.21E−06	1.33E−06
*FGFR2*	2263	1.403E+01	1.521E+01	1.731E+01	1.48E−06	5.90E−07	5.70E−07
*PDE3A*	5139	6.473E+00	1.527E+01	3.278E+01	1.19E−05	1.02E−06	4.29E−07
*PDPN*	10630	1.899E+01	1.866E+01	2.240E+01	2.14E−05	1.11E−05	1.07E−05
*SFRP2*	6423	1.534E+01	3.079E+01	3.832E+01	9.81E−07	1.80E−07	1.50E−07

**Fig. 2:**
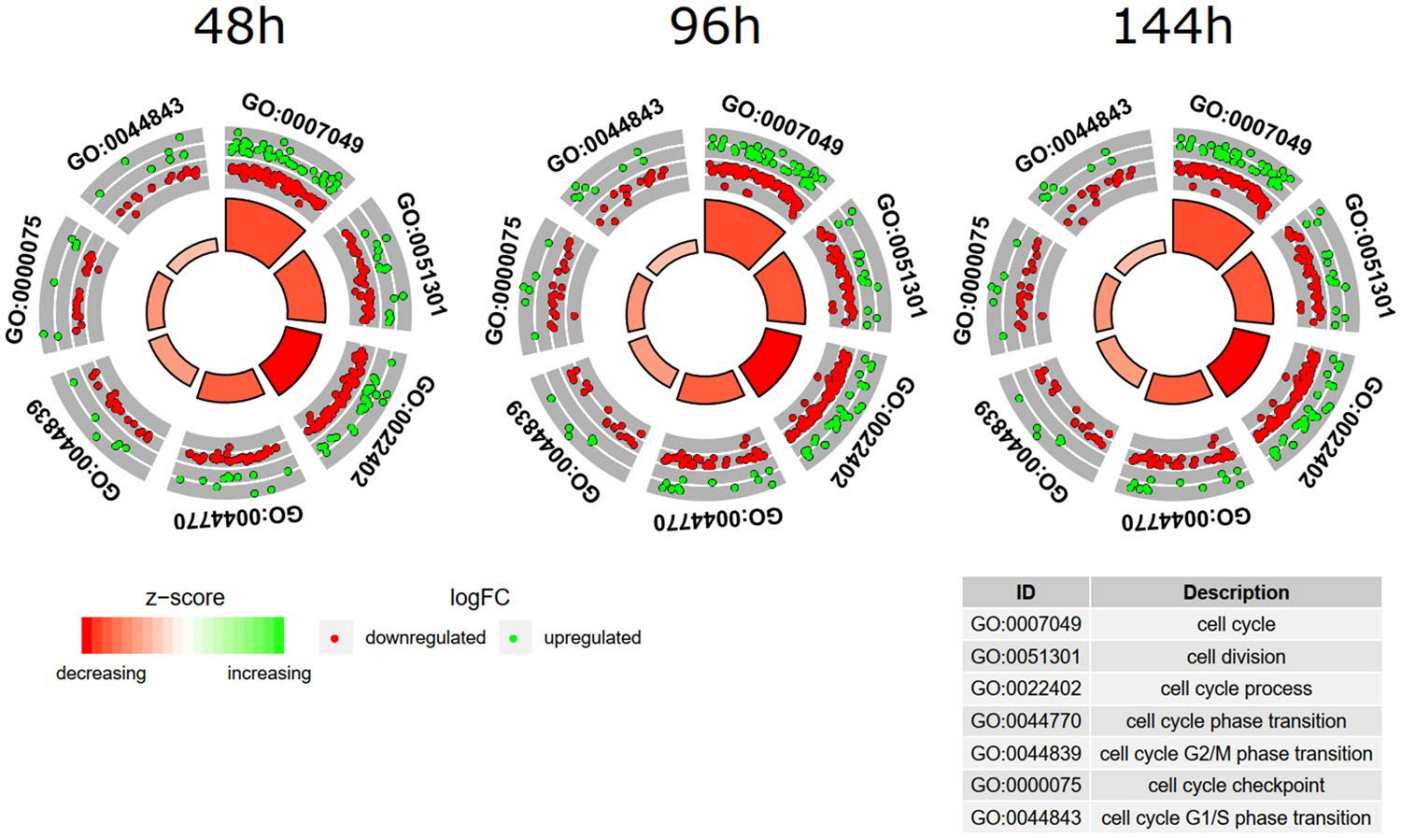
The circular visualization of the results of gene-annotation enrichment analysis. The outer circle shows a scatter plot for each term of the logFC of the assigned genes. Green circles display up-regulation and red ones down-regulation. The inner circle is the representation of *z*-score. The size and the color of the bar correspond to the value of *z*-score

To further investigate the changes within the chosen GO BP terms, we measured the enrichment levels of each selected GO BPs. The enrichment levels were expressed as *z*-scores and presented as circular visualizations (Fig. 2).

Chosen GO BP terms contained 133 differently expressed genes. Therefore, we have calculated the mean value of fold change ratio of each gene between 48, 96 and 144 h. Based on that criteria, we choose 10 most downregulated and 10 most upregulated genes for further analysis.

**Fig. 3:**
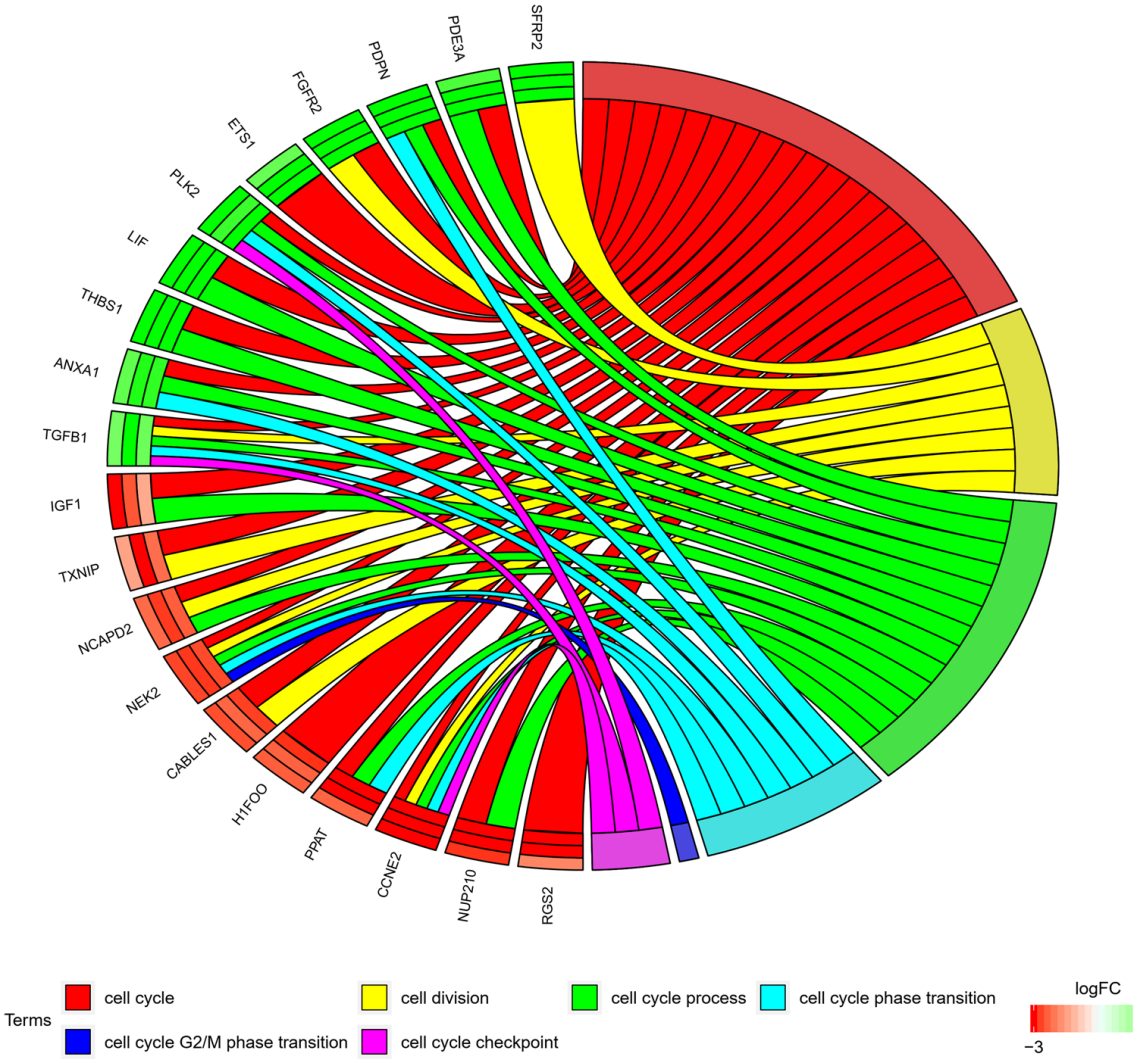
The representation of the mutual relationship between 20 chosen genes that belongs to “cell cycle checkpoint”, “cell cycle G1/S phase transition”, “cell cycle G2/M phase transition”, “cell cycle phase transition”, “cell cycle process”, “cell cycle” and “cell division” GO BP terms. The ribbons indicate which gene belongs to which categories. The colors of 3 inner bars near each gene corresponds to logFC after 48 h, 96 h and 144 h, respectively. The genes were sorted by logFC

**Fig. 4:**
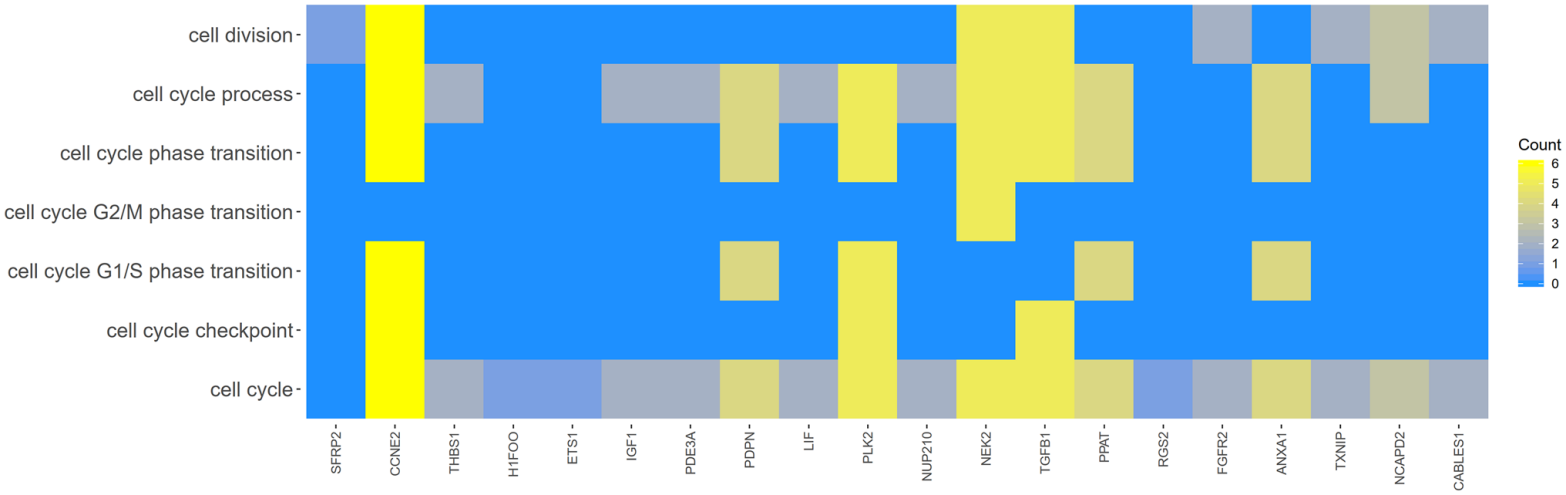
Heatmap showing the gene occurrence between 20 chosen genes that belongs “cell cycle checkpoint”, “cell cycle G1/S phase transition”, “cell cycle G2/M phase transition”, “cell cycle phase transition”, “cell cycle process”, “cell cycle” and “cell division” GO BP terms. Yellow color indicates the gene occurrence in indicated GO BP term. The intensity of colour correlates with number of GO BP Terms that selected gene belongs to

In the gene ontology database, genes that form one particular GO group can also belong to other different GO term categories. For this reason, we explore the gene intersections between the selected GO BP terms. The relation between those GO BP terms was presented as a circle plot (Fig. 3) as well as the heatmap (Fig. 4). Among the 20 chosen genes only 7 GO BP terms contain at least one gene from the chosen set.

**Fig. 5:**
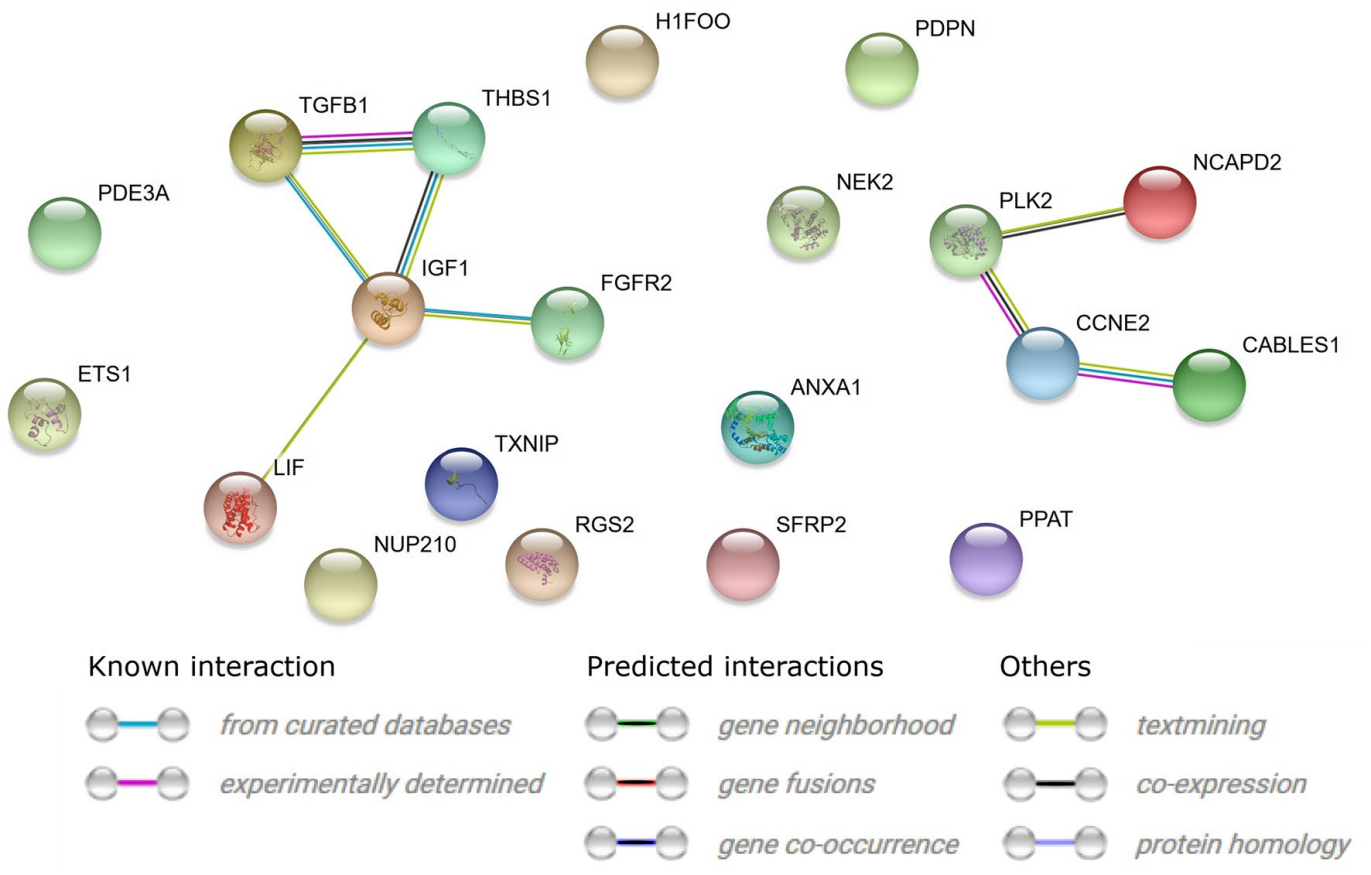
STRING-generated interaction network among 20 chosen genes belonging to the “cell cycle checkpoint”, “cell cycle G1/S phase transition”, “cell cycle G2/M phase transition”, “cell cycle phase transition”, “cell cycle process”, “cell cycle” and “cell division” GO BP terms. The intensity of the edges reflects the strength of interaction score

A STRING interaction network was generated among chosen differentially expressed genes belonging to each of the selected GO BP terms. Using such a prediction method provided us with a molecular interaction network formed between the protein products of studied genes (Fig. 5).

**Fig. 6:**
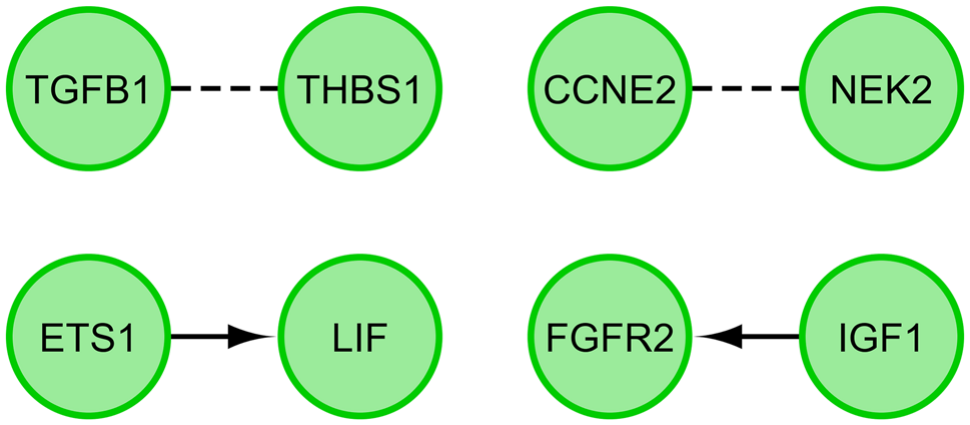
Functional interaction (FI) between 20 chosen genes that belongs to “cell cycle checkpoint”, “cell cycle G1/S phase transition”, “cell cycle G2/M phase transition”, “cell cycle phase transition”, “cell cycle process”, “cell cycle” and “cell division” GO BP terms. In the following figure “− >“ stands for activating/catalyzing, “− |” for inhibition, “–” for FIs extracted from complexes or inputs, and “—” for predicted FIs

Finally, the functional interactions between chosen genes were examined with the REACTOME FIViz app to Cytoscape 3.6.0 software. The results are shown in Fig. 6.

**Fig. 7:**
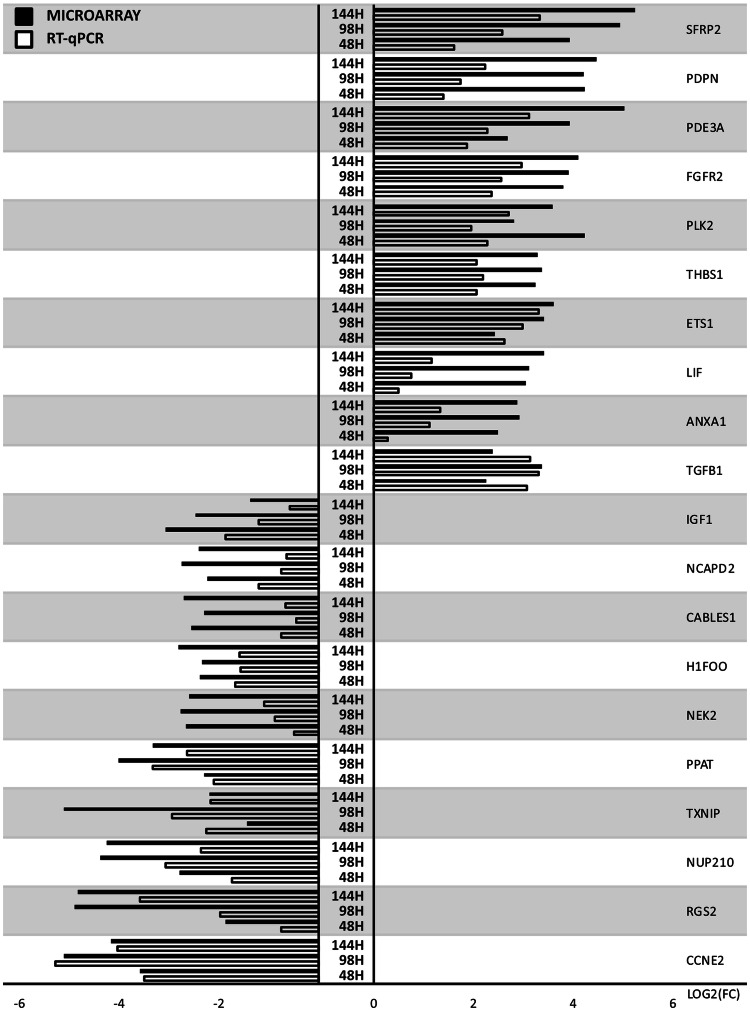
The results of RT-qPCR validation of microarray results presented in the form of a bar graph

The results of the microarray analysis were validated with the RT-qPCR methods. The obtained values were compared between both approaches and presented as a bar graph (Fig. 7).

As can be seen in Fig. 7, the direction of changes of gene expression was confirmed in all of the analyzed examples. While the scale of the changes often varies, it is understandable due to different specificity, selectivity and sensitivity of both of the methods used, with RT-qPCR tending to be much more quantitatively accurate.

Histological analysis was performed to confirm that the ovaries taken to isolate granulosa cells show the proper structure, further proofing the identity of the analyzed cells. In addition, histological image analysis enabled the observation of GCs at various stages of follicular maturation, with particular focus on mature follicles.

**Fig. 8:**
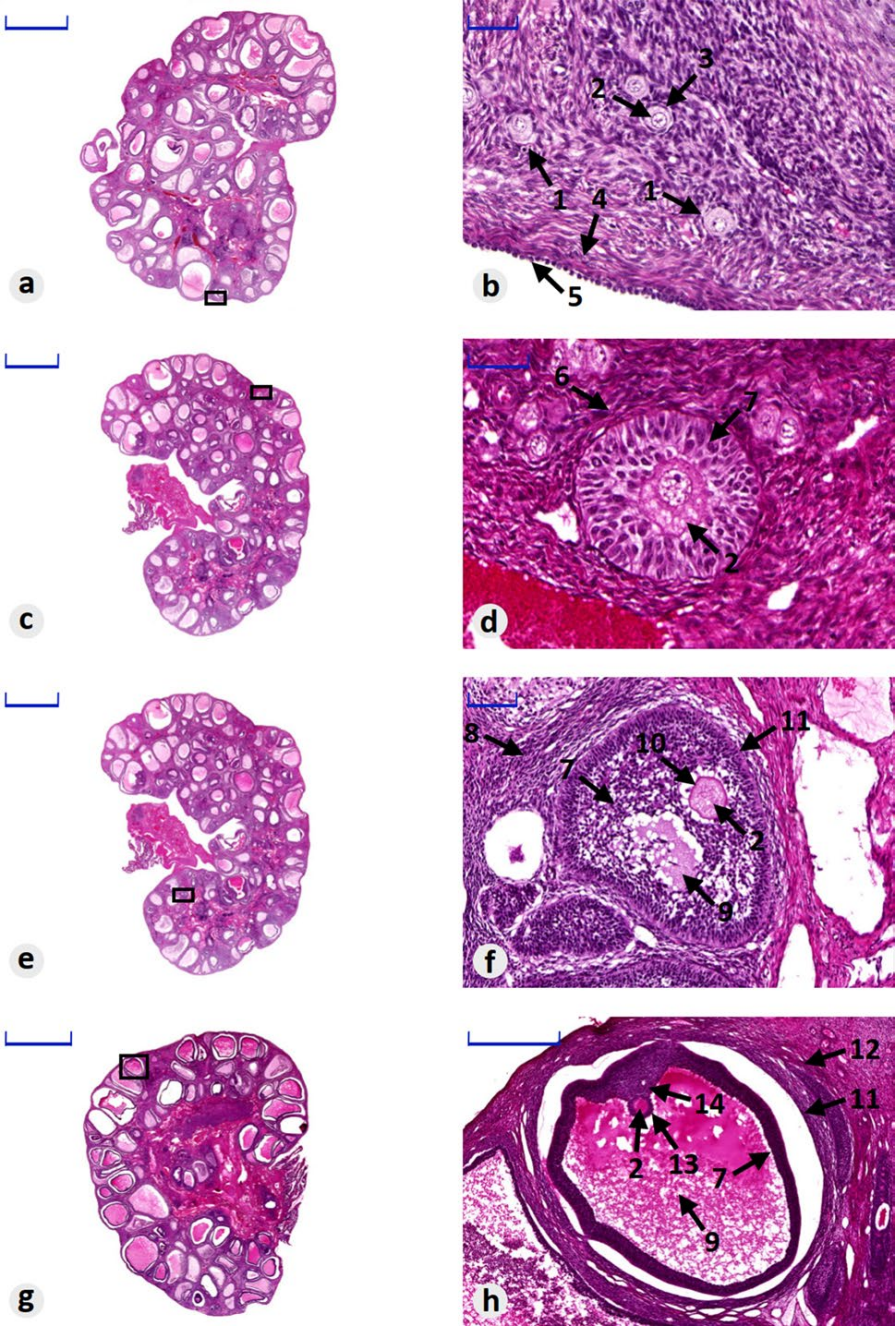
Mid-part histological sections of crossbred Landrace gilts ovaries, stained with H&E, representing their structure and follicles in all stages of development. **a**, **c**, **e**, **g** Whole ovaries (scale bars: 5000 µm), **b**, **d**, **f**, **h** selected areas of **a**, **c**, **e**, **g** observed in higher magnification (scale bars: B, D-50 µm, F-100 µm, H-500 µm). Arrows: 1—primordial follicles, 2—oocyte, 3—follicular cells, 4—tunica albuginea, 5—germinal epithelium, 6—primary follicle, 7—granulosa cells, 8—secondary follicle, 9—antrum, 10—zona pellucida, 11—theca interna and theca externa, 12—mature follicle, 13—corona radiata, 14—cumulus oophorus

Histological analysis revealed the proper structure of the collected ovaries. The organs are surrounded by tunica albuginea and the germinal epithelium. Follicles are present in the ovaries at all stages of development: numerous oocyte containing primordial follicles and one layer of flattened cells; primary follicles in which the oocyte is surrounded by 1 to many layers of granulosa cells; secondary follicles in which follicular fluid-filled space (antrum) appears between granulosa cells; and mature follicles, with an oocyte located at one of the follicle’s poles and a large antrum surrounded by granulosa cells (Fig. 8).

**Fig. 9:**
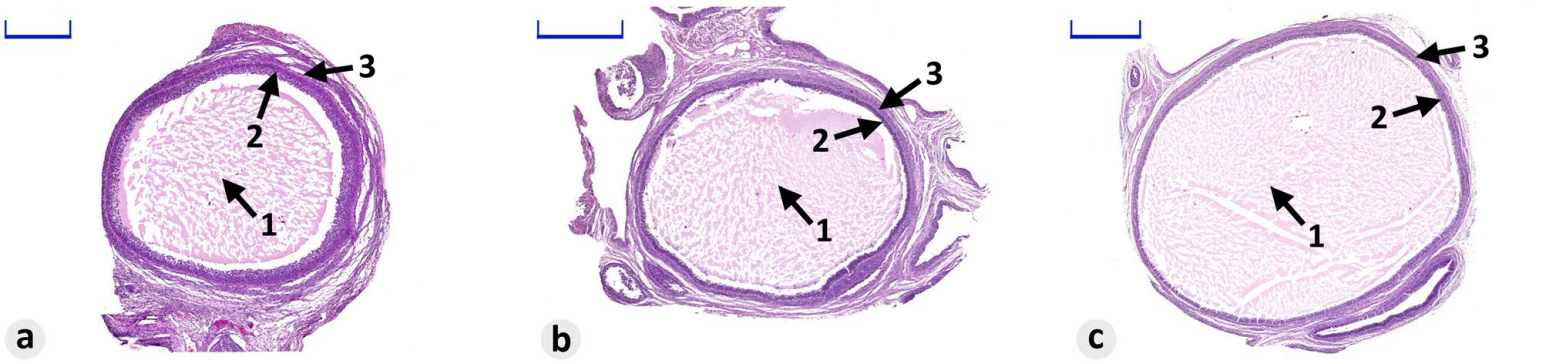
Microphotograph representing separated mature follicles classified into 3 groups according to their size (H&E staining). **a** Small follicle (< 3 mm; scale bar—500 µm), **b** medium follicle (3–5 mm; scale bar—1000 µm), **c** large follicles (> 5 mm; scale bar—1000 µm). Arrows: 1—antrum, 2—granulosa cells, 3—theca interna and theca externa

Collected separated follicles varied in size. According to the diameter of follicles, they can be classified into 3 different groups: large follicles (> 5 mm), medium (3–5 mm) and small follicles (< 3 mm) (Fig. 9).

Granulosa cells, formed during the transformation of flat cells present in primary follicles, are observed at all other stages of follicular maturation. In the primary follicles, these cells form one to several layers surrounding the oocyte (Fig. 8d-7). In the next stage, their number increases, while a cavity (antrum) filled with fluid formed at the same time (Fig. 8f-7). In a mature follicle, granulosa cells accumulate on one of the poles of follicle, forming the cumulus oophorus and corona radiata around the oocyte, as well as surrounding a fully formed follicular antrum (Fig. 8h-7, 13, 14; Fig. 9a–c-2).

## Discussion

**Fig. 10:**
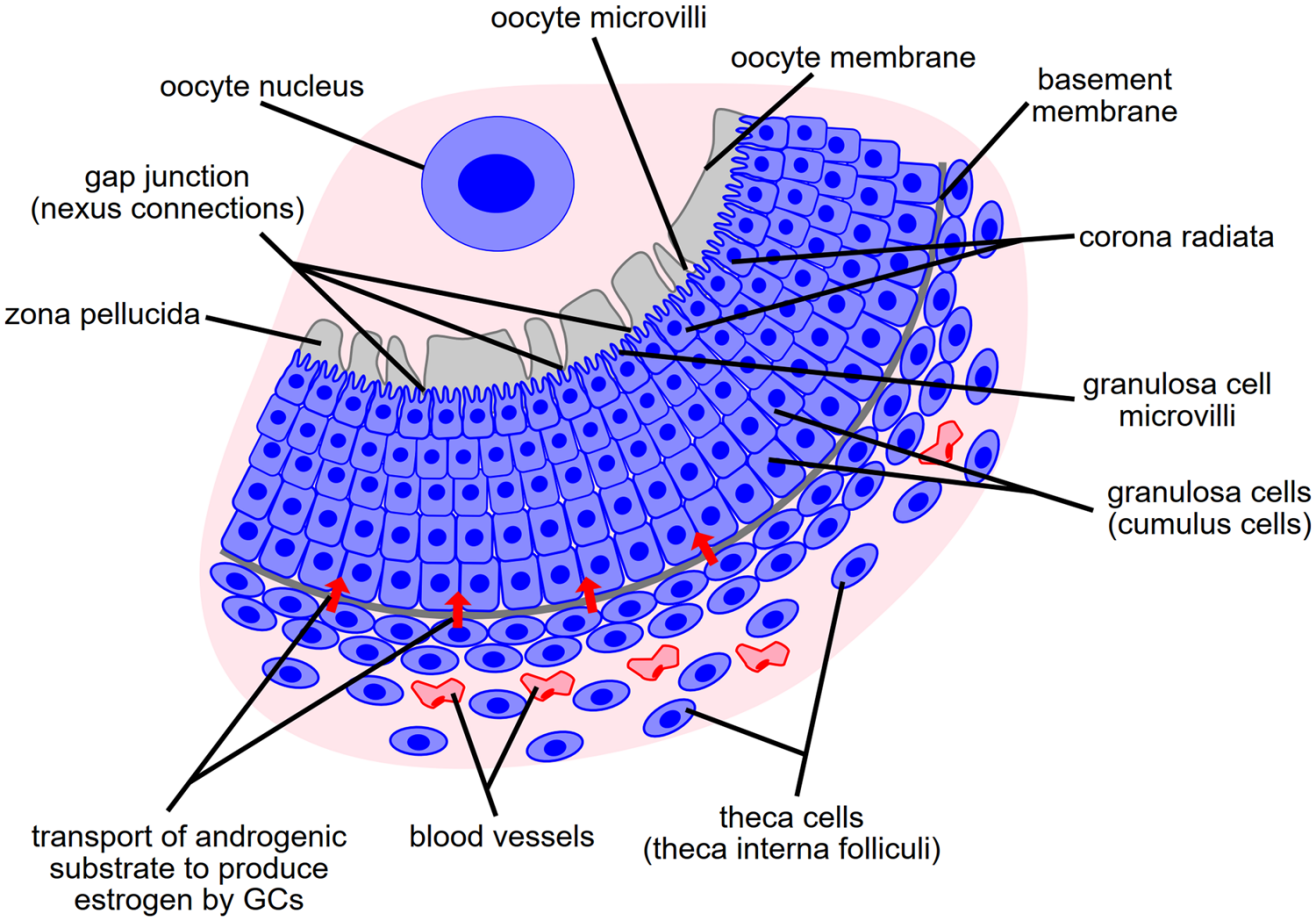
A diagram of a cross-section of the ovarian follicle, outlining its layout and features enabling functions of the distinct granulosa types

Ovarian granulosa cells (GCs) play several roles in the female ovary (Jankowski et al. 2018). By contributing to the construction of the ovarian follicle wall, these cells enable forming of the fluid-filled cavity, which provides the oocyte with an environment for development. A direct link to the oocyte is established by the CCs, namely the corona radiata, through penetration of the zona pellucida using microvilli. GCs provide the oocyte with a supply of ions, nutrients and signal molecules through gap junctions (Kempisty et al. 2013). Another activity of GCs their participation in the synthesis and secretion of steroid sex hormones. After ovulation, GCs together with theca cells fill the interior of the follicle and form a corpus luteum, producing progesterone, the key hormone responsible for maintaining pregnancy. A schematic of the ovarian wall, outlining the features necessary for the distinct function of the granulosa cells is presented on Fig. 10. A broad range of GC properties indicates their great plasticity, which reinforces the belief that the molecular background of GCs processes in in vitro culture should be thoroughly studied. They are also cells of great differentiative potential towards lineages of largely different physiological characteristics e.g. osteoblasts (Mattioli et al. 2012). GCs are a rich source of cells used for research on molecular backgrounds of the ovarian processes, which are the basis for mammalian reproduction. Granulosa can be divided into two types: mural GCs (building the wall of the follicle) and cumulus GCs (cumulus cells, CCs), which directly surround oocytes and form the *cumulus oophorus* (Rybska et al. 2018c).

The progression of the cell cycle is a sequence of successive phases in which cells perform planned actions necessary for proliferation. The checkpoint system is designed to prevent the cell from entering the next phase until the successful completion of events from the previous. Proliferation of most cells is regulated mainly in G1 phase, with some examples of its occurrence in phase G2. For example, oocytes (OC) can be retained in this phase for up to several decades until they are given the hormonal signals for further division. This is related to the oocyte development process called oogenesis. During this process, during nuclear and cytoplasmic maturation, proteins and mRNAs necessary for further development are accumulated (Budna et al. 2018).

The current study focused on measuring the level of porcine granulosa cell gene expression at the start of their primary culture (0 h) and after 48 h, 96 h and 144 h of its course. We have focused on genes belonging to seven ontological groups: “cell cycle”, “cell division”, “cell cycle process”, “cell cycle phase transition”, “cell cycle G1/S phase transition”, “cell cycle G2/M phase transition” and “cell cycle checkpoint”. Out of 133 differentially expressed genes from these GOs, we chose the 10 most up-regulated (*SFRP2*, *PDPN*, *PDE3A*, *FGFR2*, *PLK2*, *THBS1*, *ETS1*, *LIF*, *ANXA1*, *TGFB1*) and the 10 most downregulated (*IGF1*, *NCAPD2*, *CABLES1*, *H1FOO*, *NEK2*, *PPAT*, *TXNIP*, *NUP210*, *RGS2* and *CCNE2*). Almost all of the genes were members of the “cell cycle” ontological group, with the exception of the most upregulated *SFRP2*. The “cell cycle” ontological group includes the genes responsible for the progression of all biochemical and morphological events taking place in replicating cells. “Cell division” is defined as the process in which cell components are divided and partitioned to produce more cells. It is not synonymous with the term ‘cytokinesis’, which does not include the process of nuclear division. The “cell cycle process”, on the other hand, refers to a process that ensures precise and complete genome replication and chromosome segregation. “Cell cycle phase transition” references the process of cellular transition between the phases of the cell cycle. Genes participating in cell transition from G1 to S and from G2 to M are contained in the “cell cycle G1/S phase transition” and “cell cycle G2/M phase transition” GOs, respectively. The “cell cycle checkpoint” ontological group consists of genes controlling the cycle progression by monitoring the integrity of the cell after each of its phases. Their actions include detection of damage or lack of damage, often leading to downstream signal transduction.

The most up-regulated gene was *SFRP2* (*secreted frizzled-related protein 2*), which exclusively belongs to the “cell division” ontological group. It is a member of the secreted frizzled-related protein (SFRP) family, the antagonists of the WNT signaling pathways (Zamberlam et al. 2019). The WNT signaling affects the development of follicles, proliferation and differentiation of granulosa cells. This gene is commonly expressed in ovarian follicle granulosa (Hernandez-Gonzalez et al. 2006; Ekart et al. 2013). Its expression may predispose this gene to become a marker of cell division in GC in vitro culture. Another gene expressing significant up-regulation in “cell cycle”, “cell cycle process” and “cell cycle phase transition ontology groups was *PDPN* (*podoplanin*), encoding a type I integral membrane glycoprotein. Previous publications indicate its involvement in cancer-associated angiogenesis (Shindo et al. 2013). Increased expression of this gene was also demonstrated in porcine GCs (Chermuła et al. 2019). We have also observed up-regulation of *PDE3A* (*phosphodiesterase 3A*), which belongs to the “cell cycle” and “cell cycle process” GOs. Studies on in vitro matured porcine oocytes showed that PDE3A is the main PDE degrader of cAMP in oocytes. Specific inhibition of cAMP degradation by PDE3 prevents the resumption of oocyte meiosis (Sasseville et al. 2006). It also has an important role during the ovulatory gonadotropin surge (Sasseville et al. 2007). The *FGFR2* gene (*fibroblast growth factor receptor 2*), belonging to the “cell cycle” and “cell division” GOs, showed significant up-regulation during primary in vitro culture of the GCs. FGFR is a protein activated by IGF1. Its relationships with the reproductive system were indicated, among others, in the porcine umbilical cord during pregnancy (Chrusciel et al. 2011), swine endometrium (Wollenhaupt et al. 2004, 2005), swine fallopian tubes (Wollenhaupt et al. 2004) and porcine GCs (Chermuła et al. 2019). Expression of *PLK2* gene (*polo-like kinase 2*) was recorded in bovine GCs and theca cells and was recognized as a possible new genetic marker (Hatzirodos et al. 2015). Studies on rat ovaries have shown that *PLK2* is strongly involved in cell cycle processes. Excessive expression of this gene was shown to retain GCs in the G0/G1 phase (Li et al. 2012). The *PLK2* gene plays a role in normal cell division, with its expression also observed in swine buccal mucosa cells, indicating it as a marker of processes associated with that tissue (Dyszkiewicz-Konwińska et al. 2018). In our research, this gene has been shown to belong to the “cell cycle”, “cell cycle process”, “cell cycle phase transition” and “cell cycle checkpoint” ontology groups. This data indicates that PLK2 gene is a candidate marker of the cell cycle driving processes, with particular participation in checkpoint control. Another up-regulated gene is *THBS1* (*thrombospondin 1*), the increased expression of which was recorded during follicular atresia in swine GCs (Terenina et al. 2017). *ETS1* (*ETS proto*-*oncogene 1*) is a transcription factor involved in the regulation of extracellular matrix reconstruction (Garrett-Sinha 2013). Up-regulation of this gene has been observed in various human cancers, as well as the culture of porcine buccal mucosa cells (Dyszkiewicz-Konwińska et al. 2018). *LIF* (*leukaemia inhibitory factor interleukin 6 family cytokine*) plays an important role in normal cell cycle development, especially through its participation in checkpoint control. This gene was studied using porcine induced pluripotent stem cells (iPSC) and in vitro matured metaphase II oocytes (Yuan et al. 2014). Other studies analyzed the effect of porcine leukemia recombinant inhibitory factor (pLIF) on in vitro maturation of oocytes (Dang-Nguyen et al. 2014). A significant increase in the rate of maturation of oocytes in cumulus-oocyte complexes cultured with the addition of pLIF was observed, with lack of effect with the same protein was added after the removal of cumulus cells (Dang-Nguyen et al. 2014). LIF may play a role in the maturation of oocytes through regulation of the cell cycle predisposing LIF to become a genetic marker of GC in vitro culture. *ANXA1* (*annexin A1*) gene, encoding a membrane phospholipid-binding protein also exhibited upregulation along the time of culture and has a role in inhibiting phospholipase A2, as well as an anti-inflammatory effect. In humans, this gene has been shown to be associated with various types of cancer (Pessolano et al. 2018). Apoptosis of in vitro cultured swine kidney cells was observed after the addition of TNF-alpha, associated with the transfer of *ANXA1* to or around the nucleus (Ishido 2005). *TGFB1* (*transforming growth factor beta 1*) is a gene involved in many cellular processes, including tissue repair, inflammatory cell chemoattraction and angiogenesis, also known as a mediator of fibroblast–myofibroblast differentiation (Dyszkiewicz-Konwińska et al. 2018). In our study, it showed up-regulation in the “cell cycle”, “cell cycle process”, “cell cycle phase transition”, “cell cycle checkpoint” and “cell division” ontology groups. The most down-regulated gene was *CCNE2* (*cyclin E2*), which, as shown in studies on bovine GCs, is associated with proliferation of granulosa cells in particular stages of folliculogenesis (Shimizu et al. 2013). This gene is one of the key regulators of the cell cycle, with its expression recorded in mouse GCs (Meinsohn et al. 2018). The *CCNE2* gene belongs to the same ontological groups as the *TGFB1* gene described above. The regulator of G protein signaling 2 is a protein encoded by the *RGS2* gene, a known marker of GC luteinization (Kranc et al. 2015). In our research, we noted a significant down-regulation of this gene in the “cell cycle” GO. *NUP210* (*nucleoporin 210*) is a gene encoding one of the building blocks of nuclear pores, allowing the transport of molecules between the nucleus and cytoplasm. Recently, the role of this gene as a critical regulator of the muscle and neuron differentiation process has also been described (Gomez-Cavazos and Hetzer 2015). To the best of our knowledge, the expression of this gene in the cells of the porcine ovary has not yet been described. However, its expression in granulosa cells and bovine theca cells has been noted (Hatzirodos et al. 2015). In our study, *NUP210* showed downregulation in the “cell cycle” and “cell cycle process” groups. The *TXNIP* gene (*thioredoxin interacting protein*), belonging to the “cell cycle” and “cell division” groups, was downregulated in our research. The expression of this gene was observed in bovine cumulus cells (Salhab et al. 2013), porcine oocytes (Ożegowska et al. 2018), CCs (Borys et al. 2018) and porcine oviductal epithelial cells (Kulus et al. 2019a, b). It is responsible for regulating the response to oxidative stress but also contributes to glucose metabolism and lactate production. It was shown that this gene is important in the meiotic maturation process of mouse oocytes (Lee et al. 2013). Analysis of gene expression during GC short-term primary in vitro culture, also revealed a downregulation of the *PPAT* gene (*phosphoribosyl pyrophosphate amidotransferase*), which encodes a member of the family of phosphorus-pyramid transfer proteins. PPAT in cattle was closely related to the *PAICS* gene, with its location mapped to BTA6 (Bønsdorff et al. 2004). Among the analyzed genes, the only representative of the “cell cycle G2/M phase transition” ontological group was the *NEK2* (*NIMA related kinase 2*) gene. This gene also belonged to other GOs, with its downregulation observed during the culture. Earlier studies indicate its active participation in the start and progression of metaphase II in in vitro maturated porcine oocytes (Fujioka et al. 2000) suggesting potential uses of this gene as a marker of G2/M cycle phase transition in in vitro cumulus–oocyte complexes. The *H1FOO* gene (*oocyte*-*specific H1 histone*), belonging to only one ontological group (“cell cycle”), showed down-regulation in our studies. The expression of this gene is restricted to the ovary. Sheng and co-authors studied the localization of *H1FOO* in pig ovaries at different stages of postpartum (Sheng et al. 2015). The studies showed its differential presence in oocytes depending on the stage of follicle development. In GCs, weak *H1FOO* expression was observed in primordial follicles, with a moderate increase in early growing follicles, developing antral follicles and antral follicles only after 72dpp and 95dpp, respectively (Sheng et al. 2015). These studies suggest that GCs may influence oocyte development through the *H1FOO* signalling. In the current study, GCs were cultured without oocytes, which could possibly explain the downregulation of this gene. Such a close link between this gene and the ovary, as the only place of its expression and function, may predispose it as an in vitro genetic marker of ovarian cells. *CABLES1* (*Cdk5 and Abl enzyme–substrate 1*) is a gene closely related to the regulation of the cell cycle (Lee et al. 2007). Its downregulation has been recorded in “cell cycle” and “cell division” groups. The loss of *CABLES1* expression was associated with the presence of ovarian cancer in humans, with excessive expression of this gene leading to cancer cell apoptosis (Sakamoto et al. 2008). These findings prove the function of this gene as a suppressor of ovarian cancer in humans. In our studies, *NCAPD2* (*non SMC condensin I complex subunit D2*) showed downregulation, in “cell cycle”, “cell cycle process” and “cell division” GOs. This gene plays a role in the condensation of chromatin preceding cell division (Martin et al. 2016). Its expression was recorded in the ovary during studies on the resistance of human ovarian cancer cells to cisplatin (Solár and Sytkowski 2011). It was also found that mutations within this gene may have a significant effect on oncogenic processes in the mouse ovary (Emmanuel et al. 2011). The last downregulated gene was *IGF1* (*insulin-like growth factor 1*). It is associated with such important processes as cell growth, prevention of apoptosis and cell proliferation. It has been proven that it interacts with the FSH and LH hormones, influencing the proliferation of GCs (Filus and Zdrojewicz 2014). It is also believed that it increases the influence of gonadotropins on ovarian steroidogenesis (Kranc et al. 2017). Experiments on bovine and porcine ovaries have shown that IGF1 was one of the factors limiting or promoting multiple ovulation (Sirotkin et al. 2017).

Mutual correlations of the analyzed genes were analyzed using STRING-generated interaction network. Most genes (11) did not show a direct correlation with others. However, it is impossible to exclude indirect mutual regulation.

Genes whose expression in GCs was deemed to be of importance for the processes of cell cycle progression were revealed in the gene analysis. Several of these genes were earlier associated with the reproductive system, with some described for the first time in this context. The comparison of the results of this study with the research mentioned above may be the basis for the consolidation of some data on the expression of the genes of interest in the given context. However, the presence of potential new gene markers may provide a new point of reference for subsequent research. In conclusion, significant changes in the expression of the described genes and their functional participation in cell cycle processes indicate their potential role as markers of primary porcine granulosa cell in vitro cultures.
